# Comparative Efficacy of Brief Psychoanalytic Psychotherapy and Cognitive Behavioral Therapy for Generalized Anxiety Disorder: A Randomized Controlled Trial

**DOI:** 10.3390/jcm15041472

**Published:** 2026-02-13

**Authors:** Metin Çınaroğlu, Eda Yılmazer, Selami Varol Ülker, Gökben Hızlı Sayar

**Affiliations:** 1 Psychology Department, Faculty of Administrative and Social Science, İstanbul Nişantaşı University, Maslak Mah. Taşyoncası Sok. No: 1V ve No:1Y, Sarıyer, İstanbul 34398, Türkiye; 2 Psychology Department, Faculty of Social Science, Beykoz University, İstanbul 34398, Türkiye; edayilmazer@beykoz.edu.tr; 3 Faculty of Humanities and Social Sciences, Üsküdar University, İstanbul 34398, Türkiye; selamivarol.ulker@uskudar.edu.tr; 4 Psychiatry, Institute of Social Science, Medical School, Üsküdar University, İstanbul 34398, Türkiye; gokben.hizlisayar@uskudar.edu.tr

**Keywords:** generalized anxiety disorder, cognitive behavioral therapy, psychoanalytic psychotherapy, psychodynamic therapy, randomized controlled trial, brief psychotherapy

## Abstract

**Background**: Cognitive behavioral therapy (CBT) is the most established psychological treatment for generalized anxiety disorder (GAD), yet many patients do not achieve full remission. Brief psychoanalytic psychotherapy represents a theoretically distinct alternative, but direct controlled comparisons remain limited. This study examined the short-term efficacy of brief psychoanalytic psychotherapy and CBT relative to a waitlist control in adults with GAD. **Methods**: In a three-arm randomized controlled trial, 60 adults with DSM-5-diagnosed GAD were allocated to brief psychoanalytic psychotherapy (12 weekly sessions), CBT (12 weekly sessions), or a waitlist control. Assessments were conducted at pre-treatment and post-treatment. The primary outcome was anxiety severity measured by the Beck Anxiety Inventory (BAI). Secondary outcomes included depressive symptoms (BDI-II), quality of life (WHOQOL-BREF), functional impairment (WHODAS 2.0), and therapeutic alliance (Working Alliance Inventory). Data were analyzed using mixed-design ANOVAs and effect size estimates. **Results**: Both active treatments produced significantly greater reductions in anxiety than the waitlist control, with large effect sizes. Mean BAI scores decreased by 14.5 points in the psychoanalytic group and 16.3 points in the CBT group, compared to minimal change in the waitlist condition. Similar patterns were observed for depressive symptoms, quality of life, and functional impairment, with both therapies outperforming waitlist controls on all secondary outcomes. No statistically significant differences were found between CBT and brief psychoanalytic psychotherapy on any outcome measure. Therapeutic alliance ratings were high and comparable across the two active treatments. **Conclusions**: Brief psychoanalytic psychotherapy and CBT were both effective short-term treatments for GAD and superior to no treatment, with no significant differences between the two modalities at post-treatment. These findings suggest that time-limited psychoanalytic psychotherapy may represent a promising short-term therapeutic option to CBT for GAD, expanding treatment options for patients and clinicians.

## 1. Introduction

Generalized anxiety disorder (GAD) is a common and chronic anxiety condition characterized by excessive, uncontrollable worry about everyday concerns, with a lifetime prevalence of approximately 6–7% in adult populations [1]. GAD often follows a protracted course with high relapse rates and frequently co-occurs with depression and other anxiety disorders [2]. Individuals with GAD experience significant distress and impairment in social and occupational functioning, as well as reduced quality of life [3]. These features contribute to treatment challenges, as many patients require long-term management and continue to report residual symptoms following therapy [4]. Although available treatments are associated with symptom reduction, pathological worry—the core feature of GAD—often persists [5], and a substantial proportion of patients do not achieve full remission even with established interventions [6]. Given the prevalence and burden of GAD, there remains a clear need to optimize treatment approaches. Many patients prefer psychotherapy over pharmacological options, and current clinical guidelines recommend psychotherapy, particularly cognitive behavioral therapy, as a first-line treatment for moderate to severe GAD [7] Nevertheless, improving outcomes for individuals who do not respond fully to initial therapies remains an important clinical objective.

Cognitive behavioral therapy (CBT) is the most well-established psychotherapy for generalized anxiety disorder (GAD), supported by a robust empirical evidence base. Multiple randomized trials and meta-analyses have demonstrated that CBT leads to significant reductions in anxiety symptoms compared to no-treatment controls [8,9]. A systematic review of 25 studies, for example, reported that patients receiving CBT were more likely to show reduced anxiety at the end of treatment than those on waiting lists or receiving treatment-as-usual [10]. CBT has also been shown to alleviate excessive worry and comorbid depressive symptoms commonly observed in GAD. Core components of CBT for GAD include psychoeducation about anxiety and worry, cognitive restructuring of maladaptive thoughts, behavioral strategies such as exposure or applied relaxation, and interventions targeting avoidance and intolerance of uncertainty [11]. When delivered individually over approximately 10–15 sessions, CBT is associated with moderate to large improvements in anxiety severity and functional outcomes. Meta-analytic evidence further indicates that CBT outperforms placebo and other control conditions, with treatment gains often maintained at follow-up [12]. Nevertheless, a substantial proportion of patients continue to experience clinically significant worry following a standard course of CBT [13], motivating ongoing efforts to refine CBT protocols and to examine alternative or adjunctive treatment approaches.

In contrast to CBT’s symptom-focused, skills-based approach, psychodynamic psychotherapy (including psychoanalytic therapy) emphasizes the exploration of emotional conflicts and relational patterns associated with anxiety. Therapeutic techniques commonly include examination of unconscious fears, past experiences, interpersonal difficulties, and processes occurring within the therapeutic relationship, with the aim of increasing insight into factors contributing to chronic anxiety [14]. Psychodynamic therapy is widely used in the treatment of anxiety and mood disorders; however, until relatively recently, its empirical evidence base in generalized anxiety disorder (GAD) was more limited. Prior reviews have noted that comparatively few studies specifically examined short-term psychodynamic psychotherapy for GAD, despite its frequent clinical application. This gap has begun to be addressed by an increasing number of controlled studies.

Meta-analytic evidence indicates that psychodynamic therapy is associated with reductions in anxiety symptoms. A comprehensive meta-analysis of psychodynamic treatments for anxiety disorders, including 14 randomized controlled trials, reported that short-term psychodynamic psychotherapy was significantly more effective than control conditions, with a pooled effect size of approximately g ≈ 0.6 [15]. The same meta-analysis found no statistically significant differences when comparing psychodynamic therapy with other bona fide psychological treatments at post-treatment or at follow-ups of up to one year [16]. Similar patterns have been reported in studies of psychodynamic therapy for depression and related conditions.

Despite these findings, the evidence base for psychodynamic therapy in GAD remains smaller than that for CBT. Adaptations of psychodynamic techniques for GAD have been described, including a focus on themes such as fear of loss, anger, or needs for control that may be associated with persistent worry. Available case series and smaller trials have reported symptom improvement following such interventions. Nevertheless, further randomized and comparative studies are needed to more fully characterize the efficacy of psychodynamic therapy for GAD, particularly in relation to treatments with different theoretical frameworks such as CBT.

Despite the widespread use of both cognitive behavioral therapy (CBT) and psychodynamic psychotherapy in the treatment of generalized anxiety disorder (GAD), direct comparative studies remain limited. A 2007 Cochrane review of psychological therapies for GAD noted that, at that time, only one randomized trial had directly compared CBT with psychodynamic psychotherapy, highlighting a significant gap in the literature. That trial, conducted by Leichsenring et al. [17], randomized patients with GAD to either short-term psychodynamic psychotherapy or CBT, without inclusion of a waitlist control, and reported that both treatments were associated with significant and large reductions in anxiety symptoms. On the primary outcome measure, an observer-rated anxiety scale, no statistically significant difference was observed between the two treatments, indicating comparable improvements at post-treatment. These findings were supported by results from several self-report measures, which likewise showed symptom improvement in both treatment groups.

Differences between treatments were reported on certain secondary outcomes. Specifically, CBT was associated with greater reductions in trait anxiety, worry severity, and comorbid depressive symptoms compared to psychodynamic therapy. In a subsequent follow-up assessment of the same cohort, both treatment groups maintained their overall anxiety reductions at 12 months, with no significant difference in global anxiety severity, while the CBT group showed greater improvements on measures of worry and trait anxiety. The authors emphasized the need for larger and methodologically rigorous studies to further examine potential differences between treatments and to identify patient characteristics associated with differential treatment response.

Since the time of that trial, direct head-to-head studies in this area remain scarce. A few related studies have appeared—for example, an Internet-based guided self-help RCT for GAD found no short-term outcome differences between an applied relaxation/CBT program and a psychodynamic self-help program, with both yielding moderate to large symptom improvements by follow-up [18]. Another small trial in a clinical sample explored an integrative therapy combining psychodynamic and CBT techniques, and reported that this blended approach was more effective than standard CBT alone [19]. These studies, while encouraging, each had limitations such as small sample sizes or non-standard treatment formats. To date, there has not been a published randomized trial incorporating both a psychodynamic therapy and CBT arm and a no-treatment control in the same design for GAD. The absence of a control condition in prior head-to-head studies makes it difficult to determine how much improvement is due to the specific effects of therapy vs. non-specific factors or the natural course of symptoms. Furthermore, previous comparisons have generally examined full-length courses of therapy (e.g., 12–30 sessions), whereas there is growing interest in briefer interventions that could be more accessible.

Beyond modality-specific techniques, a longstanding debate in psychotherapy research concerns the relative contribution of common vs. specific factors to treatment outcomes. Common factors—such as therapeutic alliance, expectancy, and therapist competence—have been shown to account for a substantial proportion of outcome variance across different psychotherapeutic approaches, whereas specific factors refer to techniques unique to particular treatment models (e.g., cognitive restructuring in CBT or interpretive work in psychodynamic therapy). Meta-analytic and comparative studies suggest that bona fide psychotherapies often yield broadly comparable outcomes, particularly for internalizing disorders, raising questions about the mechanisms underlying observed treatment effects. Within this context, direct comparative trials can help clarify whether observed equivalence reflects shared common factors, distinct pathways leading to similar outcomes, or a combination of both.

The present study was designed to address these gaps through a three-arm randomized controlled trial comparing brief psychoanalytic psychotherapy and cognitive behavioral therapy (CBT) with a waitlist control in adults with generalized anxiety disorder (GAD). Each active treatment was delivered over 12 weekly sessions (approximately three months), reflecting a time-limited format intended to balance clinical effectiveness with practicality. Inclusion of a waitlist control enabled evaluation of treatment effects beyond changes attributable to time or repeated assessment.

The primary aim was to examine the efficacy of brief psychodynamic therapy and CBT relative to no treatment and to compare outcomes between the two active interventions. We hypothesized that both therapies would produce significantly greater reductions in anxiety symptoms, along with improvements in worry, mood, functioning, and quality of life, compared with the waitlist condition. Based on the prior literature, we anticipated that CBT might demonstrate greater effects on excessive worry, whereas brief psychoanalytic psychotherapy might yield comparable overall anxiety reduction through insight-oriented processes. However, given previous findings of broadly equivalent efficacy, no strong a priori hypothesis was made regarding superiority on the primary outcome. This trial was therefore intended to provide a rigorous parallel comparison of the two approaches and to contribute new evidence on the role of brief psychodynamic therapy within the evidence-based treatment landscape for GAD.

## 2. Methods

### 2.1. Study Design

This study employed a three-arm, parallel-group randomized controlled trial design to examine the comparative effectiveness of brief psychoanalytic psychotherapy and cognitive behavioral therapy (CBT) relative to a waitlist control condition in adults with generalized anxiety disorder (GAD). The trial was prospectively registered at ClinicalTrials.gov (NCT06831708) prior to participant enrollment. Participants were randomly allocated in equal proportions to one of the three study arms. Assessments were conducted at pre-treatment and at the end of the 12-week study period. Anxiety symptom severity, measured by the Beck Anxiety Inventory (BAI), was defined as the primary outcome. Secondary outcomes included depressive symptoms (Beck Depression Inventory–II), quality of life (WHOQOL-BREF), functional impairment (WHODAS 2.0), and the quality of the therapeutic relationship (Working Alliance measure). Changes in these outcomes over time were compared across groups to evaluate treatment effects. The present study was designed to detect clinically meaningful differences between active treatment conditions and a no-treatment (waitlist) control, rather than to establish superiority between the two active psychotherapies. Given resource and feasibility constraints, no formal a priori power calculation was conducted. With approximately 20 participants per group, the study was adequately powered to detect large between-group effects relative to the waitlist condition, as observed in prior psychotherapy trials for generalized anxiety disorder. However, the sample size was not sufficient to reliably detect small-to-moderate differences between CBT and brief psychoanalytic psychotherapy. Accordingly, the comparison between the two active treatments should be interpreted as exploratory.

### 2.2. Participants

Participants were adults diagnosed with generalized anxiety disorder (GAD) who were recruited from psychiatric outpatient settings in Istanbul, Türkiye, including a private psychiatric clinic affiliated with one of the authors psychiatry professor (GHS) and collaborating private and governmental hospital outpatient services. All participants had received a GAD diagnosis within the previous six months from a licensed psychiatrist based on DSM-5 diagnostic criteria. Diagnostic eligibility was independently confirmed prior to randomization through a structured clinical interview conducted by two authors (EY and SVÜ), both assistant professors of clinical psychology. A total of 60 participants were enrolled and randomized. During the study, five participants discontinued participation (two from the waitlist control group, one from the brief psychoanalytic psychotherapy group, and two from the CBT group). Reported reasons for dropout included health problems, family-related difficulties, demanding work schedules, or unspecified reasons.

Inclusion criteria were: (a) age 18 years or older; (b) a current primary diagnosis of generalized anxiety disorder; (c) diagnosis confirmed by both a psychiatrist and independent clinical interview; and (d) ability to participate in online, individual psychotherapy sessions and complete self-report assessments. Exclusion criteria included: (a) current psychotic disorder, bipolar disorder, or severe major depressive episode requiring immediate intervention; (b) active substance use disorder; (c) high suicide risk requiring urgent clinical care; (d) concurrent engagement in structured psychotherapy during the study period; and (e) cognitive or neurological conditions that could interfere with informed consent or study participation.

Participants were randomized in a 1:1:1 ratio to brief psychoanalytic psychotherapy, cognitive behavioral therapy, or a waitlist control using a computer-generated randomization sequence. Sequence generation was conducted by an independent researcher who was not involved in participant recruitment, eligibility assessment, treatment delivery, or outcome evaluation.

Allocation concealment was ensured through a centralized assignment procedure. The randomization list was stored in a secure digital file accessible only to the independent researcher responsible for randomization. Investigators involved in enrollment, baseline assessment, treatment delivery, and data analysis had no access to the allocation sequence at any stage.

Following completion of baseline assessments and confirmation of eligibility, the enrolling investigator notified the independent researcher, who then disclosed the participant’s group assignment directly to the treating therapist. Group allocation was revealed only after enrollment and baseline assessment were completed, ensuring concealment up to the point of assignment. Outcome data collection and statistical analyses were conducted without access to the allocation sequence.

### 2.3. Interventions

Participants assigned to the active treatment arms received 12 weekly individual psychotherapy sessions, delivered in a one-to-one online format. Each session followed a consistent duration across modalities. All four authors (three of them asst. professors in clinical psychology and one full professor in psychiatry) served as therapists, each delivering intervention corresponding authors to their formal training and expertise. Treatment fidelity was monitored throughout the study by an independent supervision and evaluation committee led by a senior professor of psychiatry, who was not involved in treatment delivery or outcome assessment. Adherence monitoring focused on consistency with core modality-specific principles rather than strict session-by-session manualization. For CBT, monitored criteria included use of structured session agendas, cognitive restructuring techniques, behavioral interventions (e.g., exposure or relaxation strategies), and homework review. For brief psychoanalytic psychotherapy, monitored criteria included maintenance of a psychoanalytic therapeutic stance, focus on affective and relational themes, use of clarification and interpretation, and attention to transference-related processes. Supervision meetings were held regularly to review session content and ensure adherence to these criteria. Overall adherence was judged to be satisfactory across therapists and treatment arms. Formal inter-rater reliability statistics were not calculated, as adherence evaluation was conducted as part of expert supervision rather than independent double rating.

**Brief Psychoanalytic Psychotherapy:** Brief psychoanalytic psychotherapy was delivered by two authors (MÇ and SVÜ), both trained clinical psychologists with advanced expertise in psychodynamic and psychoanalytic treatment approaches. The intervention was grounded in contemporary psychoanalytic theory and adapted to a time-limited format suitable for anxiety disorders. Treatment focused on identifying and working through unconscious anxiety-related conflicts, maladaptive relational patterns, and affective processes contributing to generalized anxiety symptoms. Core therapeutic techniques included exploration of recurring themes, clarification, interpretation of defenses, and examination of transference–countertransference dynamics as they emerged within the therapeutic relationship. Particular attention was given to patterns of worry, intolerance of uncertainty, and internalized relational expectations underlying chronic anxiety. Although the treatment followed shared psychoanalytic principles, it was not manualized in a rigid session-by-session format. Instead, interventions were flexibly tailored to each participant’s clinical presentation while maintaining a consistent psychoanalytic framework and therapeutic stance. The time-limited structure emphasized maintaining therapeutic focus and continuity while facilitating insight and emotional processing within the 12-session frame. Although brief psychoanalytic psychotherapy was not delivered via a rigid session-by-session manual, it followed a structured, time-limited framework consistent with contemporary short-term psychodynamic approaches. Treatment was organized into three overlapping phases: (1) an initial phase (sessions 1–3) focused on establishing the therapeutic frame, clarifying the central anxiety-related conflicts, and identifying recurring interpersonal and affective themes; (2) a middle phase (sessions 4–9) emphasizing exploration and interpretation of core relational patterns, affective experiences, and defenses associated with chronic worry and intolerance of uncertainty; and (3) a termination phase (sessions 10–12) focused on consolidation of insight, review of therapeutic themes, and processing of separation and treatment ending. While interventions were tailored to individual clinical material, therapists adhered to these core phase-specific objectives throughout treatment.

**Cognitive Behavioral Therapy (CBT):** CBT was delivered by two authors (EY and GHS), both trained in evidence-based cognitive behavioral interventions for anxiety disorders. The CBT intervention followed established treatment principles for generalized anxiety disorder and was structured, goal-oriented, and problem-focused. Treatment components included psychoeducation about anxiety and worry processes, identification and modification of maladaptive automatic thoughts and core beliefs, behavioral experiments, and strategies for reducing avoidance and excessive reassurance-seeking. Sessions also incorporated anxiety management techniques such as relaxation strategies and skills aimed at improving emotional regulation and coping with uncertainty. CBT sessions followed a structured format, including agenda setting, review of between-session experiences, active skill-based interventions, and assignment of between-session exercises when appropriate. Treatment progression was adapted to individual symptom profiles while remaining consistent with core CBT principles.

**Control Condition (Waitlist):** Participants assigned to the control condition were placed on a waitlist and did not receive structured psychotherapy during the 12-week study period. They continued any routine psychiatric care not involving formal psychotherapy and completed the same assessment schedule as participants in the active treatment arms. Following completion of post-study assessments, participants in the control group were offered access to psychotherapy services.

**Procedure:** After providing written informed consent, participants underwent pre-treatment assessment prior to group allocation. Eligibility was verified through review of psychiatric diagnoses and independent clinical interviews conducted before enrollment. Following confirmation of eligibility, participants were randomly assigned to one of the three study conditions. Participants allocated to the active treatment arms commenced psychotherapy shortly after randomization and attended weekly online sessions over a 12-week period. Those assigned to the waitlist condition did not receive structured psychotherapy during this time but completed assessments according to the same schedule.

All primary and secondary outcome measures were assessed using standardized self-report questionnaires completed electronically by participants at pre-treatment and immediately following completion of the intervention or equivalent waiting period. No independent outcome assessors were involved, as outcomes were self-administered. Given the nature of psychotherapy interventions, neither participants nor therapists could be blinded to treatment allocation. To reduce expectancy effects, participants were informed that both active treatments were considered potentially effective, and no claims of superiority were communicated. Therapists did not participate in outcome data entry or statistical analysis.

Treatment delivery and adherence were monitored throughout the study by an independent supervision committee to ensure consistency with modality-specific standards. Post-treatment assessments were completed by participants who remained in the study, after which the study period concluded. Participants were permitted to continue ongoing pharmacological treatment provided that medication type and dosage had been stable for at least four weeks prior to study entry. Initiation of new psychotropic medications, dose adjustments, or discontinuation of existing medications were not permitted during the 12-week study period. No changes in psychotropic medication were allowed during the intervention period. Medication status was recorded at baseline and monitored throughout the study to ensure stability across treatment conditions. At baseline, a proportion of participants were receiving stable psychotropic medication as part of routine clinical care. Medication use was recorded prior to randomization and monitored throughout the study to ensure stability. The most common medication classes included selective serotonin reuptake inhibitors (SSRIs), serotonin–norepinephrine reuptake inhibitors (SNRIs), and benzodiazepines prescribed on a regular or as-needed basis. No participant initiated, discontinued, or changed the dosage of psychotropic medication during the intervention period. Baseline medication use was comparable across treatment groups (see Table 1b).

### 2.4. Measures

#### 2.4.1. Beck Anxiety Inventory (BAI)

The Beck Anxiety Inventory (BAI) was used as the primary outcome measure to assess the severity of anxiety symptoms. The BAI was originally developed by Beck et al. [20] as a 21-item self-report instrument designed to measure the somatic and cognitive components of anxiety. Items are rated on a 4-point Likert scale ranging from 0 (“not at all”) to 3 (“severely”), yielding total scores between 0 and 63, with higher scores indicating greater anxiety severity. The Turkish adaptation and validation of the BAI was conducted by Ulusoy et al. [21] in psychiatric outpatient samples. The Turkish version demonstrated excellent internal consistency (Cronbach’s *α* = 0.93) and satisfactory item–total correlations, supporting its reliability and construct validity in clinical populations. In the present study, the BAI was selected as the primary outcome due to its sensitivity to change in anxiety-focused interventions and its strong psychometric support in Turkish samples. BAI was selected as the primary outcome measure because it is a widely used, well-validated instrument for assessing overall anxiety severity and has demonstrated sensitivity to treatment-related change in generalized anxiety disorder. Although the BAI includes a substantial somatic component, prior clinical trials in GAD have successfully used it to capture global anxiety symptom reduction, particularly in intervention studies comparing active treatments with control conditions. In the present study, the primary objective was to evaluate short-term changes in overall anxiety severity rather than to isolate pathological worry as a single symptom dimension. Measures of worry-specific outcomes were therefore considered secondary in importance, and the absence of a dedicated worry instrument is acknowledged as a limitation.

#### 2.4.2. Beck Depression Inventory–II (BDI-II)

Depressive symptom severity was assessed using the Beck Depression Inventory–II (BDI-II). The BDI-II was developed by Beck et al. [22] as a revision of the original BDI, aligned with DSM-IV diagnostic criteria for major depressive disorder. The instrument consists of 21 items rated on a 4-point scale (0–3), with total scores ranging from 0 to 63. The Turkish validity and reliability study of the BDI-II was conducted by Kapçı et al. [23] in both clinical and nonclinical adult samples. The Turkish version demonstrated high internal consistency (Cronbach’s *α* = 0.89–0.90), strong test–retest reliability (r = 0.94), and satisfactory convergent and discriminant validity. These findings support the use of the BDI-II as a reliable measure of depressive symptoms in Turkish psychiatric populations. In the present study, the BDI-II was included as a secondary outcome to capture changes in comorbid depressive symptoms frequently observed in individuals with GAD.

#### 2.4.3. World Health Organization Quality of Life—Brief Version (WHOQOL-BREF)

Quality of life was evaluated using the World Health Organization Quality of Life—Brief Version (WHOQOL-BREF). The WHOQOL-BREF was developed by the World Health Organization as a cross-culturally applicable measure of subjective quality of life. It consists of 26 items assessing four domains: physical health, psychological health, social relationships, and environmental quality of life [24]. The Turkish version of the WHOQOL-BREF was developed within the WHO collaborative framework and has demonstrated acceptable reliability and validity in Turkish populations [25]. Reported internal consistency coefficients for domain scores generally range from acceptable to good (*α* values typically > 0.70). The WHOQOL-BREF was included as a secondary outcome to assess broader treatment-related changes in perceived well-being beyond symptom reduction.

#### 2.4.4. World Health Organization Disability Assessment Schedule 2.0 (WHODAS 2.0)

Functional impairment and disability were assessed using the World Health Organization Disability Assessment Schedule 2.0 (WHODAS 2.0). WHODAS 2.0 was developed by the World Health Organization to provide a standardized measure of disability across physical and mental health conditions, replacing the Global Assessment of Functioning in DSM-5-related contexts [26]. The scale assesses functioning across six domains: cognition, mobility, self-care, getting along, life activities, and participation. The Turkish validity and reliability study of the WHODAS 2.0 was conducted by Aslan et al. [27] in psychiatric patients and healthy controls. The 36-item self-report version demonstrated satisfactory to high internal consistency in clinical samples (Cronbach’s *α* values generally ≥ 0.80) and strong evidence of construct and criterion validity. In the present study, the WHODAS 2.0 was used as a secondary outcome to capture changes in everyday functioning associated with psychotherapy.

#### 2.4.5. Working Alliance Inventory (WAI—Short Form)

The quality of the therapeutic relationship was assessed using the Working Alliance Inventory (WAI). The original WAI was developed by Horvath and Greenberg [28] based on Bordin’s (1979) pan-theoretical model of the therapeutic alliance, which conceptualizes alliance in terms of agreement on goals, agreement on tasks, and the emotional bond between therapist and client. The Turkish adaptation and short-form validation were conducted by Gülüm et al. [29]. The Turkish WAI Short Form consists of 12 items and has demonstrated strong internal consistency, with Cronbach’s *α* values of 0.86 for the client form and 0.90 for the therapist form. The scale has shown good construct validity and is widely used in Turkish psychotherapy research. In this study, the WAI was included to examine the therapeutic process and alliance quality across treatment modalities. WAI was included as a process outcome to assess the quality of the therapeutic relationship. Consistent with its conceptualization as a treatment process variable, the WAI was administered only to participants in the active treatment groups and was assessed at post-treatment only, following completion of the 12-session intervention.

### 2.5. Statistical Analysis

All statistical analyses were conducted using IBM SPSS Statistics (version 30; IBM Corp., Armonk, NY, USA). Descriptive statistics (means, standard deviations, frequencies) were calculated to summarize sociodemographic and pre-treatment clinical characteristics. Pre-treatment equivalence across the three groups was examined using one-way analyses of variance (ANOVA) for continuous variables and chi-square tests for categorical variables. Primary and secondary outcomes were analyzed using mixed-design analyses of variance, with time (pre-treatment, post-treatment) as the within-subjects factor and group (brief psychoanalytic psychotherapy, CBT, waitlist control) as the between-subjects factor. The primary hypothesis concerned the group × time interaction, indicating differential change over time across treatment conditions. When significant interaction effects were observed, post hoc comparisons with appropriate correction for multiple testing were conducted to identify between-group differences. Analyses were performed using a complete-case approach, including participants who completed post-treatment assessments. Effect sizes were calculated to estimate the magnitude of treatment effects, with partial eta squared (*η*^2^*p*) reported for ANOVA effects and standardized mean differences used for pairwise comparisons where relevant. All statistical tests were two-tailed, and the significance level was set at α = 0.05. Assumptions of normality and homogeneity of variance were evaluated prior to inferential analyses. Where necessary, robustness of results was confirmed through inspection of residuals and variance patterns. For outcomes where lower scores indicate improvement (BAI, BDI-II, WHODAS 2.0), negative Cohen’s d values indicate better post-treatment outcomes in the first-listed group. Analyses were conducted using a complete-case approach rather than an intention-to-treat (ITT) framework because post-treatment outcome data were unavailable for participants who discontinued the study, limiting the feasibility of standard ITT implementations based on observed change scores. Although alternative strategies for handling missing data (e.g., mixed-effects models or conservative imputation) are possible, these were not implemented given the low attrition rate and the balanced distribution of dropouts across groups. Attrition was low (5 of 60 participants) and occurred across all study arms. Given the limited extent of missing data and the short study duration, complete-case analysis was retained as the primary analytic strategy. Although a complete-case approach was used for the primary analyses, alternative analytic strategies for handling missing data are available, including mixed-effects models for repeated measures or conservative imputation approaches. Given the very low attrition rate (8.3%), the balanced distribution of dropouts across groups, and the large observed between-group effects relative to the waitlist control, the primary conclusions are unlikely to be materially altered by reasonable assumptions about missing data. Nevertheless, the absence of formal sensitivity analyses represents a limitation, and future studies with larger samples should incorporate ITT-based modeling approaches to further assess robustness.

### 2.6. Registered vs. Reported Outcomes

The primary and secondary outcome measures reported in this manuscript were pre-specified in the registered protocol for this trial (ClinicalTrials.gov Identifier: NCT06831708). The primary outcome was change in overall anxiety severity measured by the Beck Anxiety Inventory (BAI) from baseline to immediately post-intervention. Registered secondary outcomes included depressive symptoms (Beck Depression Inventory-II), quality of life (WHOQOL-BREF), functional impairment (WHODAS 2.0), quality of the therapeutic alliance (Working Alliance Inventory), and overall treatment satisfaction. Outcomes and assessment time points (baseline and post-treatment) are consistent with the registration entry, and no deviations from the registered analysis plan for these primary or secondary outcomes were made in the present manuscript.

## 3. Results

### 3.1. Participant Flow and Attrition

**Figure 1:**
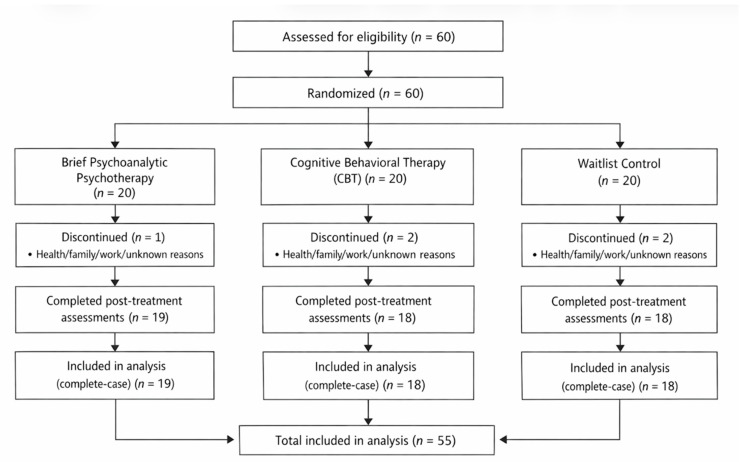
CONSORT Flow Diagram.

A total of 60 participants were randomized to brief psychoanalytic psychotherapy, cognitive behavioral therapy (CBT), or a waitlist control condition. Five participants discontinued participation during the study period (two from the waitlist group, one from the psychoanalytic psychotherapy group, and two from the CBT group). Reported reasons for dropout included health-related issues, family circumstances, demanding work schedules, or unspecified reasons. A descriptive comparison indicated that participants who discontinued did not differ meaningfully from completers in baseline sociodemographic characteristics or baseline symptom severity. All remaining participants completed post-treatment assessments. Participant flow is summarized in the CONSORT diagram (Figure 1).

Figure 1 outlines the number of participants at each stage, including enrollment (*n* = 60), allocation to brief psychoanalytic therapy (*n* = 20), CBT (*n* = 20), or waitlist control (*n* = 20), follow-up, and analysis. A total of 5 participants (2 waitlist, 1 psychoanalytic, 2 CBT) discontinued participation, yielding an analyzed sample of N = 55 completers.

### 3.2. Pre-Treatment Characteristics

Pre-treatment sociodemographic and clinical characteristics were comparable across groups (Table 1a). There were no significant between-group differences at pre-treatment in age (*p* = 0.81), gender distribution (*p* = 0.93), or any pre-treatment outcome measure (BAI, BDI-II, WHOQOL-BREF, WHODAS 2.0; all *p* > 0.8), indicating successful randomization (Table 1a).

In Table 1a, data are presented as mean ± SD for continuous variables and *n* (%) for categorical variables. No significant pre-treatment differences were found between the psychoanalytic, CBT, and waitlist groups in age, gender distribution, or any pre-treatment outcome measure (all *p* > 0.80).

Psychotropic medication doses were stable for at least four weeks prior to randomization and remained unchanged throughout the 12-week study period. Percentages may exceed 100% because some participants were prescribed more than one medication class.

### 3.3. Primary Outcome: Anxiety Symptoms (BAI)

**Figure 2:**
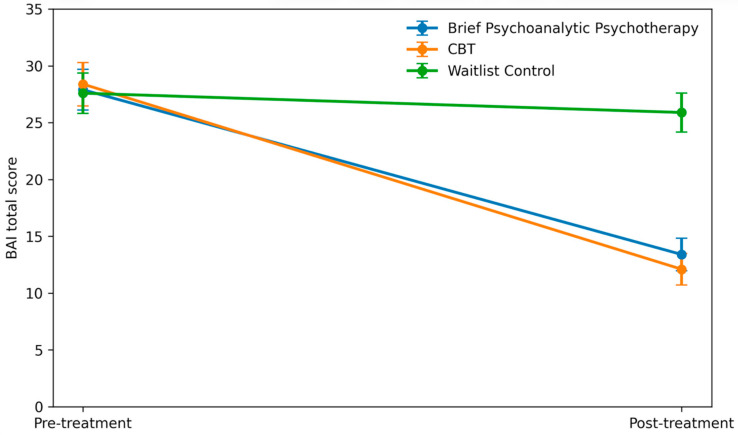
Changes in Anxiety Symptoms.

There was a significant group × time interaction for anxiety severity, *F*(2, 52) = 23.94, *p* < 0.001, *η*^2^*p* = 0.47, indicating differential change in BAI scores between conditions. Mean BAI scores declined substantially from pre-treatment (~28 points in each group) to post-treatment in both therapy conditions, whereas the waitlist group showed minimal change. In the brief psychoanalytic psychotherapy group, BAI decreased from 27.9 (SD = 7.8) at pre-treatment to 13.4 (6.2) at post-treatment, a mean reduction of –14.5 points. Similarly, the CBT group mean fell from 28.4 (8.1) to 12.1 (5.9), a reduction of –16.3 points. In contrast, the waitlist group’s BAI was essentially unchanged (27.6 (7.5) at pre-treatment vs. 25.9 (7.3) at post, Δ = −1.7). Post hoc comparisons confirmed that both active treatments produced significantly greater anxiety reduction than waitlist (*p* < 0.001 for each). The between-group effect sizes for anxiety improvement were large for psychoanalytic therapy vs. waitlist (*d* = −1.85, 95% CI [−2.62, −1.08]) and for CBT vs. waitlist (*d* = −2.08, 95% CI [−2.89, −1.27]). There was no significant difference in anxiety outcomes between the two active therapies (*p* = 0.67), with a small effect size favoring CBT (*d* ≈ 0.21). Mean BAI scores at each time point and change scores are presented in Table 2, and the reduction in anxiety symptoms by group is illustrated in Figure 2. The post-treatment BAI difference was large for psychoanalytic therapy vs. waitlist (*d* = −1.85, 95% CI [−2.62, −1.08]) and for CBT vs. waitlist (*d* = −2.08, 95% CI [−2.89, −1.27]), while the psychoanalytic therapy vs. CBT difference was small (*d* = 0.21, 95% CI [−0.43, 0.86]).

In Table 2, mean (SD) BAI scores at pre-treatment and post-treatment are shown for each group, along with the mean change (Δ) from pre-treatment. Both brief psychoanalytic psychotherapy and CBT groups exhibited large decreases in BAI scores from pre- to post-treatment, whereas the waitlist group showed minimal change.

In Figure 2, values are mean BAI scores at pre-treatment and post-treatment for the brief psychoanalytic psychotherapy, CBT, and waitlist control groups. Both active treatments show marked reductions in anxiety symptoms from pre- to post-treatment, whereas the waitlist group exhibits minimal change. Error bars represent standard deviations. Note: Both brief psychoanalytic therapy and CBT achieved significantly greater anxiety reduction than waitlist (*p* < 0.001).

### 3.4. Secondary Outcomes

All secondary outcome measures exhibited a pattern of significant improvement in the two treatment groups relative to the waitlist control.

#### 3.4.1. Depressive Symptoms (BDI-II)

**Figure 3:**
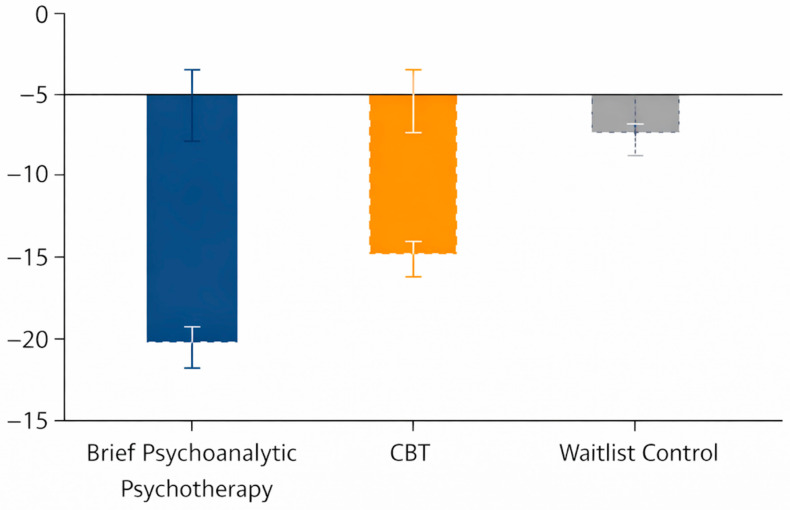
Changes in Depressive Symptoms (Beck Depression Inventory-II) by Group.

A significant group × time interaction was found for depressive symptoms, *F*(2, 52) = 8.03, *p* = 0.001, partial *η*^2^ = 0.22. Both brief psychoanalytic therapy and CBT led to marked decreases in BDI-II scores from pre- to post-treatment, whereas the waitlist group showed no meaningful change. The psychoanalytic group’s mean BDI-II score dropped from 21.6 (SD = 8.9) at pre-treatment to 11.8 (7.1) post-treatment (Δ = −9.8 points), and the CBT group’s mean fell from 22.1 (9.3) to 10.9 (6.8) (Δ = −11.2). By contrast, the waitlist group’s mean BDI-II was 21.2 (8.6) at pre-treatment and 19.8 (8.4) at post (Δ = −1.4). Both active treatments yielded significantly greater reductions in depressive symptoms than the waitlist condition (*p* < 0.005 for each), with large between-group effect sizes for psychoanalytic psychotherapy vs. waitlist (*d* = −1.03, 95% CI [−1.72, −0.34]) and for CBT vs. waitlist (*d* = −1.16, 95% CI [−1.87, −0.46]). There was no significant difference between the two therapies on depression improvement (*p* = 0.78), with a negligible effect size (*d* ≈ 0.13). Outcome data for BDI-II are provided in Table 3, and Figure 3 depicts the changes in depression scores by group. The post-treatment BDI-II difference was large for psychoanalytic therapy vs. waitlist (*d* = −1.03, 95% CI [−1.72, −0.34]) and for CBT vs. waitlist (*d* = −1.16, 95% CI [−1.87, −0.46]), while the psychoanalytic therapy vs. CBT difference was small (*d* = 0.13, 95% CI [−0.52, 0.77]).

In Table 3, mean (SD) BDI-II scores at pre-treatment and post-treatment are provided for each group, as well as the mean change from pre-treatment. The psychoanalytic and CBT groups demonstrated considerable reductions in depressive symptoms compared to the waitlist group, which had little change.

In Figure 3, displayed are mean BDI-II scores at pre-treatment and post-treatment for the psychoanalytic therapy, CBT, and waitlist groups (lower scores indicate fewer depressive symptoms). Both active treatments show substantial decreases in depressive symptoms over time compared to the negligible change observed in the waitlist condition. Error bars represent standard deviations. Note: Both brief psychoanalytic therapy and CBT resulted in significantly greater depression improvement than waitlist (*p* < 0.005).

#### 3.4.2. Quality of Life (WHOQOL-BREF)

Quality of life improved significantly in both treatment groups compared to the control, as reflected in a significant group × time interaction *F*(2, 52) = 9.02, *p* = 0.001, *η*^2^ = 0.25. Participants receiving psychoanalytic therapy or CBT showed increases in WHOQOL-BREF total scores, whereas the waitlist group’s quality of life remained essentially stable. The psychoanalytic group’s WHOQOL-BREF total score rose from 61.3 (SD = 9.7) at pre-treatment to 73.6 (10.1) post-treatment (Δ = +12.3), and the CBT group’s score increased from 60.8 (10.2) to 75.1 (9.4) (Δ = +14.3). The waitlist group showed only a minimal change (61.9 (9.4) at pre-treatment vs. 63.1 (9.6) at post; Δ = +1.2). Both therapy groups demonstrated significantly greater quality-of-life gains than waitlist (*p* < 0.005 for each). The between-group effect sizes were large (*d* ≈ 1.06 for psychoanalytic vs. waitlist; *d* ≈ 1.26 for CBT vs. waitlist). No significant difference was detected between psychoanalytic therapy and CBT on WHOQOL-BREF improvement (*p* = 0.60, *d* ≈ 0.15). Table 4 summarizes the quality of life outcomes by group and time. The post-treatment WHOQOL-BREF total score difference favored psychoanalytic therapy vs. waitlist (*d* = 1.06, 95% CI [0.38, 1.75]) and CBT vs. waitlist (*d* = 1.26, 95% CI [0.55, 1.98]), while the psychoanalytic therapy vs. CBT difference was small (*d* = −0.15, 95% CI [−0.80, 0.49]).

In Table 4, mean (SD) overall quality of life scores at pre-treatment and post-treatment are presented for each group, with the mean score change from pre-treatment. Both active treatment conditions showed improved quality of life relative to pre-treatment, whereas the waitlist group’s quality of life remained largely unchanged.

#### 3.4.3. Functional Impairment (WHODAS 2.0)

Functional disability scores (WHODAS 2.0) showed a significant group × time interaction as well *F*(2, 52) = 17.21, *p* < 0.001, *η*^2^ = 0.40. Both active treatments were associated with substantial reductions in self-reported functional impairment compared to minimal change in the waitlist group. In the psychoanalytic therapy arm, the mean WHODAS score declined from 2.31 (SD = 0.54) at pre-treatment to 1.48 (0.49) post-treatment (Δ = −0.83), and in the CBT arm from 2.36 (0.58) to 1.41 (0.46) (Δ = −0.95). The waitlist group’s mean WHODAS changed only from 2.28 (0.51) to 2.15 (0.50) (Δ = −0.13). Improvements in functioning were significantly greater in both therapy groups compared with the waitlist condition (*p* < 0.001 for each), with large between-group effect sizes for psychoanalytic psychotherapy vs. waitlist (*d* = −1.35, 95% CI [−2.07, −0.64]) and for CBT vs. waitlist (*d* = −1.54, 95% CI [−2.28, −0.80]). There was no significant functional outcome difference between the two therapies (*p* = 0.71; *d* ≈ 0.15). Detailed results for functional impairment are reported in Table 5. The post-treatment WHODAS 2.0 total score difference was large for psychoanalytic therapy vs. waitlist (*d* = −1.35, 95% CI [−2.07, −0.64]) and for CBT vs. waitlist (*d* = −1.54, 95% CI [−2.28, −0.80]), while the psychoanalytic therapy vs. CBT difference was small (*d* = 0.15, 95% CI [−0.50, 0.79]).

In Table 5, mean (SD) WHODAS scores at pre-treatment and post-treatment are shown for each group, along with mean change from pre-treatment. Lower scores indicate less disability. Both the psychoanalytic therapy and CBT groups experienced substantial functional improvement (decreased disability) compared to the waitlist group, which showed minimal change in functioning.

#### 3.4.4. Therapeutic Alliance (Working Alliance Inventory)

The quality of the therapeutic alliance, measured by the Working Alliance Inventory, was high in both active treatment conditions and did not differ significantly between the two therapies. Mean WAI total scores (measured on a 1–7 scale) were 5.92 (SD = 0.58) in the psychoanalytic group and 5.87 (0.61) in the CBT group at the end of treatment (Table 6). There was no significant between-group difference in alliance ratings (*p* = 0.68), with a very small effect size (*d* = 0.08) favoring the psychoanalytic arm. Similarly, alliance subscale scores (bond, task, and goal agreement) were comparable between the two treatments (all *p* > 0.5, Table 6). Notably, alliance was not assessed in the waitlist group (no therapy provided). These results indicate that both interventions achieved a strong therapeutic alliance of similar quality. The between-treatment difference in WAI total score was small (*d* = 0.08, 95% CI [−0.56, 0.73]).

In Table 6, mean (SD) WAI total and subscale scores are shown for the brief psychoanalytic psychotherapy and CBT groups at the end of treatment. Higher scores indicate a stronger therapeutic alliance. There were no significant differences between the two therapies on total alliance or any subscale (all *p* > 0.5). WAI was not assessed for the waitlist control group (no therapy delivered).

## 4. Discussion

This randomized controlled trial provides evidence that both brief psychoanalytic psychotherapy and cognitive-behavioral therapy are effective short-term treatments for generalized anxiety disorder and are superior to no treatment. Over the 12-week intervention period, participants in both active treatment groups showed marked reductions in anxiety symptoms compared to the waitlist control, which exhibited minimal change. Mean Beck Anxiety Inventory scores in the psychodynamic and CBT groups decreased by over 50%, corresponding to a shift from the moderate-to-severe range at pre-treatment to mild symptom levels at post-treatment, whereas anxiety levels in the waitlist group remained largely unchanged (see Section 3).

In addition to reductions in anxiety, both therapies were associated with improvements across secondary outcomes. Participants receiving either psychotherapy reported significant decreases in depressive symptoms (Beck Depression Inventory-II scores) relative to the control group, as well as gains in functional ability and overall well-being, reflected in improved quality of life (WHOQOL-BREF) and reduced functional impairment (WHODAS 2.0). When directly comparing the two active treatments, no statistically significant differences were observed across anxiety, depression, functioning, or quality-of-life outcomes at post-treatment. Thus, within the limits of the present study and the acute treatment phase, brief psychodynamic psychotherapy and CBT demonstrated comparable short-term outcomes for GAD. These findings suggest that time-limited psychotherapeutic interventions grounded in different theoretical frameworks may yield similar short-term benefits, offering multiple evidence-based treatment options for patients.

Our findings are broadly consistent with prior studies examining cognitive behavioral therapy (CBT) and psychodynamic psychotherapy for anxiety disorders. In particular, the absence of a statistically significant difference between brief psychodynamic therapy and CBT in the present trial parallels the results reported by Leichsenring et al. [17], who likewise found no significant difference in primary anxiety outcomes between CBT and a longer-course psychodynamic therapy in generalized anxiety disorder. In that study, both modalities were associated with substantial improvements in anxiety and depressive symptoms by treatment completion, a pattern comparable to the pre–post changes observed here.

Similarly, a meta-analytic review by Keefe and colleagues [15] reported that short-term psychodynamic psychotherapy was not significantly different from other active psychological treatments, including CBT, in the treatment of anxiety disorders at post-treatment and short-term follow-up. The present findings are compatible with this literature in the specific context of GAD and within a three-arm randomized design including a no-treatment control.

At the same time, prior research has identified potential differences between treatments on specific symptom dimensions. For example, Leichsenring et al. [17] observed greater reductions in pathological worry and trait anxiety following CBT, despite comparable overall anxiety improvement. In the current study, no significant between-group differences were detected for worry-related outcomes at post-treatment; however, worry was assessed indirectly through broader anxiety and quality-of-life measures rather than with a dedicated instrument such as the Penn State Worry Questionnaire. In addition, the modest sample size and brief treatment duration may have limited sensitivity to detect smaller or domain-specific differences. It therefore remains possible that differential effects between therapeutic approaches could emerge with longer follow-up periods, larger samples, or more targeted outcome measures, which could not be evaluated in the present trial.

Our results are also consistent with findings from a recent network meta-analysis of psychotherapies for adult generalized anxiety disorder, which reported strong efficacy for cognitive behavioral therapy and indicated that several other structured interventions (e.g., relaxation-based approaches and certain third-wave CBT variants) are also effective, while evidence for psychodynamic therapy, although positive, was derived from a smaller number of trials. The present study contributes to this literature by providing data from a direct comparison of brief psychodynamic psychotherapy, CBT, and a waitlist control within a single randomized design.

The inclusion of a no-treatment control group facilitates interpretation of treatment-related effects relative to symptom change over time, with both active interventions showing greater improvement than the waitlist condition. This three-arm design extends earlier two-arm comparisons that lacked a control condition. In addition, prior studies using Internet-delivered self-help formats for GAD have reported no short-term outcome differences between CBT-based and psychodynamic interventions, with both associated with symptom improvement at follow-up. The current findings are broadly compatible with these results in a clinician-guided, time-limited treatment context.

Evidence from integrative treatment trials combining CBT and psychodynamic elements has suggested potential advantages relative to single-modality CBT; however, the present study did not examine combined or sequential approaches. Consequently, while different therapeutic models may emphasize distinct intervention targets, the extent to which specific techniques or shared therapeutic factors contribute to observed outcomes cannot be determined from the current data. The comparable short-term outcomes observed here may therefore reflect the influence of common factors, modality-specific mechanisms, or a combination of both.

One key common factor examined in the present study was the therapeutic alliance, defined as the collaborative bond between patient and therapist. Working alliance was assessed in both active treatment conditions, and mean alliance ratings were high by the end of treatment in both groups (mean WAI scores approximately 5.9 out of 7), with no statistically significant difference between CBT and brief psychodynamic psychotherapy. These findings indicate that participants reported comparable levels of trust and collaboration with their therapists across modalities, despite differences in therapeutic orientation.

The formation of a strong therapeutic alliance within a brief, 12-session framework is consistent with prior psychotherapy research and is notable given the differing emphases of CBT and psychodynamic approaches. The extensive empirical literature has demonstrated that therapeutic alliance is robustly associated with treatment outcomes across a wide range of psychotherapies, independent of specific techniques. Accordingly, the high alliance ratings observed in both treatment arms provide important contextual information regarding the treatment process.

In the present study, alliance ratings did not differ meaningfully between treatment modalities, and both interventions were associated with comparable short-term outcomes. However, because alliance was not examined as a mediator or predictor of outcome, no causal conclusions can be drawn regarding its role in symptom change. The current findings therefore suggest that strong therapeutic alliances can be established in both brief CBT and brief psychodynamic psychotherapy, while the specific contribution of alliance to treatment effects remains an important topic for future investigation.

## 5. Clinical Implications

A particularly salient aspect of this study is the brevity of the interventions. We deliberately utilized a 12-week (approximately 3-month) course for both therapies to test whether meaningful change could be achieved in a short time frame. The positive results are encouraging for clinical practice. Lengthy, open-ended therapy can be a barrier for many GAD patients—some may not have the resources or time to commit to many months of weekly sessions, and healthcare systems are often strained in terms of providing long-term psychotherapy. Demonstrating that substantial improvements in anxiety, mood, and functioning can occur within a 12-session protocol has practical significance. It suggests that time-limited therapy, whether CBT or psychodynamic, can be a viable option for treating GAD, increasing the accessibility of care. From a health services perspective, effective brief treatments can allow more patients to be treated in a given timeframe and potentially at lower cost. Our trial shows that even therapies that traditionally might be delivered over a longer duration (psychoanalytic psychotherapy often extends for dozens of sessions in usual practice) can be compressed into a brief format without loss of efficacy, provided they are structured and manual-guided. This adds to evidence from controlled studies that brief psychotherapies can yield clinically significant outcomes in anxiety disorders. That said, clinicians should remain mindful that “brief” does not suit every case—GAD patients with very severe or complex presentations might still require longer or more intensive treatment. Moreover, while our findings confirm short-term efficacy, they do not address maintenance of gains. In routine practice, booster sessions or continuation therapy (or adjunct pharmacotherapy) might be needed to sustain improvements. Nonetheless, our data support the idea that starting with a short course of therapy is reasonable, and if patients respond well, they may not need more prolonged treatment. Brief CBT for GAD is already an established approach, and our study implies that a brief psychodynamic therapy could be offered as an alternative for patients who prefer a psychodynamic approach or for whom CBT is contraindicated or unavailable. For example, some individuals may have tried CBT in the past without full success and might respond better to a different approach focusing on emotional insight. The availability of empirically supported brief psychodynamic therapy broadens the toolkit for treating GAD in a patient-centered way, acknowledging that one size may not fit all.

## 6. Limitations

Several limitations of this trial should be considered when interpreting the results. First, the sample size was relatively modest (N = 60, approximately 20 participants per group). Although this was sufficient to detect large between-group effects of active treatments relative to the waitlist control, the study was likely underpowered to reliably detect small-to-moderate differences between the two active psychotherapeutic interventions. Consequently, the absence of statistically significant differences between brief psychoanalytic psychotherapy and CBT should be interpreted cautiously, as subtle but potentially clinically meaningful effects may not have been detectable. Larger, adequately powered trials will be required to more definitively examine differential efficacy between active treatments.

Second, the conclusions of the present study are limited to short-term outcomes, as no follow-up assessments beyond the immediate post-treatment period were conducted. Given the chronic and often relapsing course of generalized anxiety disorder, the durability of treatment effects and the possibility of delayed or emerging differences between therapeutic approaches could not be evaluated. Although prior research suggests that both CBT and psychodynamic therapy can yield sustained benefits at longer follow-up intervals, and that CBT may better maintain certain outcomes such as worry reduction, these longer-term effects could not be assessed within the present design.

Third, all outcomes were assessed exclusively via self-report questionnaires. While well-validated instruments were used (BAI, BDI-II, WHOQOL-BREF, WHODAS 2.0), reliance on self-report introduces inherent risks of response bias. Participants’ symptom ratings may have been influenced by expectations regarding treatment effectiveness, demand characteristics, or a desire to report improvement, particularly in the context of psychotherapy trials where therapeutic engagement is salient.

Importantly, these risks are compounded by the fact that interventions were delivered by study authors. Although therapists were not involved in outcome data entry or statistical analyses, the absence of independent outcome assessors and the lack of blinding of participants and therapists introduce unavoidable risks of performance bias, expectancy effects, and investigator allegiance bias. These factors are not eliminated by acknowledgment alone and may have influenced both the magnitude of observed effects and the apparent equivalence between treatment modalities. Accordingly, the findings should be interpreted as preliminary comparative evidence rather than definitive proof of equivalence between brief psychoanalytic psychotherapy and CBT.

Fourth, generalizability is limited. The sample consisted of adults without severe psychiatric comorbidities who were willing to engage in online psychotherapy, and the study was conducted at a single academic center. Results may therefore not generalize to individuals with more complex clinical presentations, inpatient populations, or those with limited access to digital platforms. In addition, all interventions were delivered via videoconferencing. While evidence supports the effectiveness of teletherapy for anxiety disorders, some elements of in-person treatment—particularly non-verbal and affective cues relevant to psychodynamic work—may not have been fully replicated. Thus, the present findings pertain specifically to structured, time-limited psychotherapies delivered in an online format.

Although no formal a priori power calculation was conducted, it is useful to consider the approximate sensitivity of the study for interpreting null findings between the two active treatments. With approximately 18–19 participants per active treatment arm, the study was sufficiently powered to detect large between-group effects (Cohen’s d ≳ 0.9–1.0) at conventional significance levels, but it was underpowered to reliably detect small-to-moderate differences (e.g., d ≈ 0.3–0.5) between CBT and brief psychoanalytic psychotherapy. Accordingly, the absence of statistically significant differences between the two active interventions should not be interpreted as evidence of equivalence, but rather as an indication that any true differences, if present, are likely smaller than the effect sizes detectable with the current sample. Larger trials will be required to more precisely estimate and compare the relative efficacy of active treatments.

## 7. Future Directions

This study opens several avenues for future research. An immediate next step would be to conduct a larger, multi-site trial to replicate these findings and provide a more definitive comparison of CBT and psychodynamic therapy for GAD. A sufficiently powered study could clarify whether there are small effect size differences favoring one approach on certain outcomes (for example, cognitive-oriented outcomes for CBT or perhaps interpersonal outcomes for psychodynamic therapy). It would also allow investigation of moderators of treatment response—characteristics that might make an individual more suited to one treatment or the other. Potential moderators to examine include patient preference, personality variables (such as degree of introversion, or attachment style), pre-treatment severity of worry, and presence of comorbid personality disorders. Understanding who benefits most from CBT vs. psychodynamic therapy could enable personalized treatment matching in the future. Additionally, including follow-up assessments is critical. Future studies should track patients for at least 6–12 months after treatment to see how durable the effects are and whether one therapy confers longer-lasting protection against relapse. If, for instance, CBT’s gains plateau after the active phase but psychodynamic therapy patients continue to improve in certain domains due to ongoing insight development, that would be important to know; conversely, if both groups maintain gains similarly, that reinforces the interchangeability of these approaches in terms of sustained benefit. Long-term follow-up could also shed light on whether some patients switch from response to non-response status (relapse) or vice versa after treatment, and whether any post-therapy booster or maintenance strategies are needed.

Moreover, research should delve into the mechanisms of change in each therapy. Our trial, like many comparative studies, focused on outcomes rather than process. Incorporating mediational analyses could help answer how these therapies reduce anxiety. For CBT, changes in maladaptive beliefs, intolerance of uncertainty, or worry behavior could be measured; for psychodynamic therapy, changes in insight, reduction in interpersonal conflicts, or improvements in emotion regulation could be potential mediators. Collecting such data would require appropriate tools (e.g., session questionnaires, intermediate assessments) and theoretical models for each approach. This line of research can also explore the role of common factors—for instance, measuring therapeutic alliance at multiple time points (early, mid, late therapy) could determine if alliance predicts outcome in one or both treatments, and whether enhancing alliance might especially boost outcomes in one modality. The therapeutic relationship itself could be studied in more depth; qualitative interviews might reveal different patient experiences between the two therapies despite similar quantitative alliance scores.

Another interesting direction is to test sequential or combined treatment approaches. Given evidence that an integrative therapy (mixing CBT and psychodynamic elements) may yield superior outcomes, one could investigate whether a sequential approach—say, a brief course of CBT followed by a brief course of psychodynamic therapy, or vice versa—provides added value for GAD. Perhaps patients who only partially improve with one approach could substantially benefit from switching to the other, addressing remaining facets of the disorder. For example, a patient might learn cognitive and behavioral coping skills in CBT, then transition to psychodynamic sessions to work through deeper issues that were unmasked once acute anxiety receded. Comparative effectiveness research could determine if such sequencing outperforms a single-modality course or treatment-as-usual. Additionally, future studies might consider active control conditions (such as a credible placebo psychotherapy or a generalized supportive therapy) to better isolate the specific ingredients of CBT and psychodynamic therapy. While a waitlist control was ethical and useful here, an active control would help parse how much improvement is due to the specific techniques (exposure, cognitive restructuring, interpretation of unconscious conflict, etc.) versus general therapeutic factors.

From a public health perspective, it would also be worthwhile to evaluate these therapies’ acceptability and feasibility in real-world settings. Our trial showed that drop-out rates were low and patients engaged well with both treatments (likely reflecting good acceptability), but research in routine clinical practice—including group formats or guided self-help versions—could extend the impact. Notably, group CBT and other delivery formats have shown efficacy for GAD; it remains to be tested whether a group-based brief psychodynamic therapy could similarly help GAD patients and how it compares cost-effectively.

## 8. Conclusions

This randomized trial provides evidence that a brief course of psychoanalytic (psychodynamic) psychotherapy and CBT were associated with comparable short-term improvements in anxiety, depressive symptoms, and functional impairment relative to a waitlist control in adults with generalized anxiety disorder. Within the limits of the present study, both approaches produced meaningful symptom reduction over a 12-week period, suggesting that structured, time-limited psychotherapeutic interventions grounded in different theoretical frameworks may yield similar short-term outcomes under controlled conditions.

Rather than establishing equivalence or definitive comparative efficacy, these findings contribute preliminary comparative evidence and highlight the potential role of brief psychodynamic psychotherapy as a short-term treatment option alongside CBT. The observed improvements may reflect a combination of shared therapeutic factors, such as the therapeutic alliance, as well as modality-specific techniques. However, given the modest sample size, absence of independent outcome assessors, reliance on self-report measures, lack of follow-up, and use of a complete-case analytic approach without formal sensitivity analyses, the results should be interpreted cautiously.

Further research using larger samples, independent assessment, intention-to-treat-based modeling strategies, and longer follow-up periods is needed to more precisely evaluate comparative efficacy, durability of effects, and potential moderators of treatment response. Such studies will be essential for informing more tailored and evidence-informed treatment selection for individuals with generalized anxiety disorder.

## Figures and Tables

**Table 1:** (**a**) Pre-treatment Sociodemographic and Clinical Characteristics of the Analyzed (Complete-Case) Sample by Group. (**b**) Baseline Pharmacotherapy Characteristics.

(**a**)				
**Variable**	**Psychoanalytic (*n* = 19)**	**CBT (*n* = 18)**	**Waitlist (*n* = 18)**	***p***
Age, years (M ± SD)	34.7 ± 9.1	36.2 ± 8.7	35.1 ± 9.4	0.81
Female, *n* (%)	12 (63.2%)	11 (61.1%)	12 (66.7%)	0.93
BAI (pre-treatment)	27.9 ± 7.8	28.4 ± 8.1	27.6 ± 7.5	0.91
BDI-II (pre-treatment)	21.6 ± 8.9	22.1 ± 9.3	21.2 ± 8.6	0.94
WHOQOL-BREF (total)	61.3 ± 9.7	60.8 ± 10.2	61.9 ± 9.4	0.88
WHODAS 2.0 (total)	2.31 ± 0.54	2.36 ± 0.58	2.28 ± 0.51	0.86
(**b**)				
**Medication Variable**	**Psychoanalytic**	**CBT**	**Waitlist**	***p***
Any psychotropic medication	11 (57.9%)	10 (55.6%)	9 (50.0%)	0.88
SSRIs	8 (42.1%)	7 (38.9%)	7 (38.9%)	0.97
SNRIs	3 (15.8%)	3 (16.7%)	2 (11.1%)	0.91
Benzodiazepines (PRN or regular)	5 (26.3%)	4 (22.2%)	4 (22.2%)	0.93
No medication	8 (42.1%)	8 (44.4%)	9 (50.0%)	0.88

**Table 2:** Changes in Anxiety Symptoms (BAI) by Group and Time.

Group	Pre-Treatment (M ± SD)	Post-Treatment (M ± SD)	Δ Change
Psychoanalytic	27.9 ± 7.8	13.4 ± 6.2	−14.5
CBT	28.4 ± 8.1	12.1 ± 5.9	−16.3
Waitlist	27.6 ± 7.5	25.9 ± 7.3	−1.7

**Table 3:** Changes in Depressive Symptoms (BDI-II) by Group and Time.

Group	Pre-Treatment (M ± SD)	Post-Treatment (M ± SD)	Δ Change
Psychoanalytic	21.6 ± 8.9	11.8 ± 7.1	−9.8
CBT	22.1 ± 9.3	10.9 ± 6.8	−11.2
Waitlist	21.2 ± 8.6	19.8 ± 8.4	−1.4

**Table 4:** Quality of Life Outcomes (WHOQOL-BREF Total Score).

Group	Pre-Treatment (M ± SD)	Post-Treatment (M ± SD)	Δ Change
Psychoanalytic	61.3 ± 9.7	73.6 ± 10.1	+12.3
CBT	60.8 ± 10.2	75.1 ± 9.4	+14.3
Waitlist	61.9 ± 9.4	63.1 ± 9.6	+1.2

**Table 5:** Functional Impairment (WHODAS 2.0 Total Score).

Group	Pre-Treatment (M ± SD)	Post-Treatment (M ± SD)	Δ Change
Psychoanalytic	2.31 ± 0.54	1.48 ± 0.49	−0.83
CBT	2.36 ± 0.58	1.41 ± 0.46	−0.95
Waitlist	2.28 ± 0.51	2.15 ± 0.50	−0.13

**Table 6:** Therapeutic Alliance (WAI Short Form).

Measure	Psychoanalytic (*n* = 19)	CBT (*n* = 18)	*p*
WAI Total Score (M ± SD)	5.92 ± 0.58	5.87 ± 0.61	0.68
Task subscale	5.81 ± 0.62	5.84 ± 0.65	0.79
Goal subscale	5.88 ± 0.59	5.91 ± 0.57	0.83
Bond subscale	6.06 ± 0.54	5.86 ± 0.63	0.21

## Data Availability

De-identified participant-level outcome data, summary-level datasets used for statistical analyses, and additional methodological materials (including intervention descriptions and fidelity monitoring criteria) are available from the corresponding author upon reasonable request. Materials that could compromise participant confidentiality or therapist privacy are not publicly shared.

## Clinical Trial Registration Statement

This randomized controlled trial was prospectively registered at ClinicalTrials.gov (Identifier: NCT06831708).
